# Cuticular Hydrocarbon Polymorphism in a Parasitoid Wasp

**DOI:** 10.1007/s10886-022-01401-2

**Published:** 2023-01-27

**Authors:** Tamara Pokorny, Joachim Ruther

**Affiliations:** Institute of Zoology, University of Regensburg, Regensburg, Germany

**Keywords:** Cuticular lipids, variability, chemotype, mate recognition, *Tachinaephagus zealandicus*

## Abstract

Cuticular hydrocarbons (CHCs) are major constituents of the cuticular lipid layer of insects. They serve not only as a barrier to desiccation, but often additionally mediate communication at close range. The compositions of the CHC profiles, i.e., the specific compounds and their relative amounts, usually differ between species. Additional intraspecific variability can be found between different populations, between colonies and castes of social insects, and between the sexes. Thus, such groups can often be distinguished based on distinctive compounds and/or specific compound ratios. The CHC profile may further be influenced by biotic and abiotic factors, which therefore can impact, e.g., nestmate recognition or mate choice. However, consistent intrasexual variation seems to be rare. Here, we investigated a case of intrasexual CHC variability within a single population of a parasitoid wasp. While wasps of both sexes produced the same set of compounds, the relative amounts of specific compound classes revealed the presence of intrasexual chemical phenotypes. This is, to our knowledge, the first report of three distinct female CHC profile patterns within a population of a solitary insect that uses CHCs for mate recognition. Additionally, male CHC profiles, while overall very similar, could be separated into two chemotypes by multivariate analysis. The study of species exhibiting such intraspecific and intrasexual CHC variation will advance our understanding of the effects of CHC variability on both, desiccation resistance and intraspecific communication.

**Supplementary Information:**

The online version contains supplementary material available at 10.1007/s10886-022-01401-2.

## Introduction

Chemical communication is ubiquitous in the animal kingdom (Wyatt 2014). The olfactory sense is widely studied in insects, where it is used for a variety of purposes, from sexual communication to social interactions (Greenfield 2002). Many of these interactions take place at close range, and are in such cases often mediated by cuticular lipids which cover the insects’ surface as a protective layer (Lockey 1985; Howard and Blomquist 2005; Blomquist and Bagnères, 2010). The overall composition of the lipid layer can include alcohols, ketones, or acetates, however, the predominant compounds are usually hydrocarbons (Lockey 1980, 1985). Cuticular hydrocarbons (CHCs) are typically present in species-specific combinations of compounds and their relative amounts (e.g., Bagnères and Wicker-Thomas 2010; Pokorny et al. 2015; Guillem et al. 2016), which is the basis for their use in communication (Blomquist and Bagnères, 2010). There is, however, a certain degree of intraspecific variability in CHC profiles. For example, colony and caste identity in social insects is often reflected by differing compound ratios of shared compounds (Martin et al. 2008a, b; Leonhardt et al. 2016). In general, the composition of CHCs (presence/absence and relative amounts of CHCs) can be influenced by factors such as food source (Liang and Silverman 2000; Geiselhardt et al. 2012; Kühbandner et al. 2012) or temperature/climate (Menzel et al. 2017b; Rajpurohit et al. 2021). In many species, CHC profile variation can be found between populations (geographic variation: e.g., Bonelli et al. 2015; Peña-Carillo et al. 2021). Furthermore, the CHCs used in sexual communication usually differ between the sexes in their presence and/or quantity (sex dimorphic CHC profiles, reviewed in Thomas and Simmons 2008), and may additionally reflect sexual maturity (Arienti et al. 2010; Butterworth et al. 2020). Thus, CHC variation can influence communication, e.g., adding to the distinctiveness of colony profiles used for nestmate recognition in social insects (Liang and Silverman 2000) or affecting mate choice (Geiselhardt et al. 2012).

However, consistent intrasexual CHC polymorphism that is not caused by factors as those listed above seems to be rare. In *Drosophila melanogaster* and *D. simulans*, female CHC profiles can be assigned to two CHC phenotypes that differ between populations of Sub-Saharan Africa/the Carribean (*D. melanogaster*) or West Africa (*D. simulans*) and most other populations of the respective species worldwide (Luyten 1982; Jallon and Pechine 1989). Within single populations of non-eusocial hymenopteran species, two co-occurring female CHC phenotypes have been found for *Euglossa dilemma* (Saleh et al. 2021), *Odynerus spinipes* (Wurdack et al. 2015; Moris et al. 2021), and *Philanthus triangulum* (Strohm et al. 2008a, 2010), while female *Trichrysis cyanea* have recently been reported to exhibit three different female CHC profiles. However, for the latter species it is as yet unclear whether the observed polymorphism is consistent or simply reflects the females’ reproductive state (Fröhlich et al. 2022). Despite the observed female CHC polymorphisms, males seemed to exhibit only a single CHC phenotype within a given population in three of these species (Wurdack et al. 2015; Moris et al. 2021; Saleh et al. 2021; Fröhlich et al. 2022). Male *P. triangulum*, however, showed a CHC dimorphism that was similar to that observed for females, but less pronounced (Kroiss et al. 2006; Strohm et al. 2008b ; Strohm, pers. comm.). Such cases of intra-population CHC polymorphism can lead to discoveries of interesting facets of the chemical ecology of the respective species. For example, in *O. spinipes*, the polymorphism of female CHC phenotypes (different “chemotypes”) was suggested to have evolved in response to chemical mimicry by cleptoparasites (Wurdack et al. 2015). It is unknown whether the CHCs are used for intraspecific communication in either of the four species, although in the case of *E. dilemma*, CHCs other than the chemotype-characteristic ones were found to be associated with female dominance status and reproductive physiology (Saleh et al. 2021).

The present study investigates an interesting case of intrasexual CHC polymorphism in the parasitoid wasp *Tachinaephagus zealandicus* (Hymenoptera, Encyrtidae). These wasps lay several eggs into final instar larvae of filth flies, which, after pupation, are consumed by the developing parasitoids. After a temperature-dependent development time of around three to six weeks the wasps emerge from the fly puparia (Johnston and Tiegs 1921; Ferreira de Almeida et al. 2002). Preliminary chemical analyses of CHC profiles revealed an extent of variability in the relative amounts of different compound classes that indicated the existence of distinct female CHC chemotypes. One of these is similar to the male CHC profiles (Jungwirth et al. 2021), as was the case for *O. spinipes* (Wurdack et al. 2015). Interestingly, the investigation of sexual communication in *T. zealandicus* revealed that the wasps use female CHCs for mate recognition despite the similarity of the studied male and female CHC profiles (Jungwirth et al. 2021). The presence of three female CHC chemotypes could therefore entail an intriguing complexity of the sexual communication in this species. Laying the foundation for further investigations, the present study covered three objectives: (1) to characterize and delimit the female chemotypes, (2) to certify single species identity (chemotypes not representing cryptic species), and (3) to investigate potential male CHC profile variation in *T. zealandicus*.

## Methods and Materials

### Insects

Wasps were obtained using rat carcass traps (n = 3 traps, one each on three separate, successive trapping occasions in Gießen, Germany, see Jungwirth et al. 2021). Fly larvae and pupae were removed from the carcass remains after 2 to 3.5 weeks and kept at 25 °C until fly emergence ceased. Thereafter, remaining pupae were placed singly into small petri dishes (5.7 cm diameter, 1.4 cm height, lid without ventilation) until parasitoid emergence. From each host, one to four of the emerged females were placed singly in 1.5 ml reaction tubes (equipped with several small holes in the lid for ventilation), and were allowed to oviposit for a minimum of 24 h on two to three third-instar larvae of *Lucilia caesar* to start laboratory matriline cultures. Thereafter, females were frozen in labelled reaction tubes at -21 °C.

### Extracts and Compound Analysis – F0 and F1 Females

To extract cuticular lipids, females were placed singly in 250 µl glass vial inserts positioned in 1.5 ml glass sample vials. They were extracted in 20 µl of dichloromethane for five to eight minutes before being removed from the solvent with cleaned forceps. Vials were sealed and the samples were subjected to chemical analysis by coupled gas chromatography/mass spectrometry (GC/MS, see below). Compound determination of CHCs was based on diagnostic ions, linear retention indices and the results of dimethyl disulfide derivatization (see Jungwirth et al. 2021). The first analysis comprised one female per host that had been obtained from the carcass traps (n = 290, chosen randomly from all females that had emerged from the respective host). Six peaks differing clearly in their relative abundances between the suspected CHC chemotypes were selected for analysis: 6,9-tricosadiene and 9-tricosene (together constituting one overlapping peak), 7-tricosene (second peak), 11-methyltricosane, 9-methyltricosane and 7-methyltricosane (together constituting the third peak), 6,9-pentacosadiene, 9-pentacosene (together constituting the fourth peak), 7-pentacosene (fifth peak), 13-methylpentacosane, 11-methylpentacosane, 9-methylpentacosane and 7-methylpentacosane (together constituting the sixth peak). The focal peaks were manually integrated for each chromatogram using GCMS Postrun Analysis 4.45 (Shimadzu Corporation, Kyoto, Japan). As the main difference between the CHC profile variants was found to lie in the relative ratios of specific compound classes, the integration of peaks constituted by unsaturated hydrocarbons was adjusted to comprise only either mono- or diunsaturated hydrocarbons for further analysis. Thus, comparisons between CHC profiles were based on the ratios of alkadienes, alkenes, and methylbranched alkanes. Separation of the overlapping alkadienes and alkenes in the first and the fourth peak was accomplished by overlaying the chromatograms with the fragment chromatograms of the respective molecule ions. Molecule ion chromatograms were displayed with adjusted display factors (based on representative mass spectra of the respective unsaturated hydrocarbons, Table S1), which had been determined to best reflect the actual compound ratios. Peak integration start and end points were then adjusted to encompass only either the alkadiene or both alkenes for each respective chain length. The alkadiene was integrated singly by setting the end point of peak integration to the point at which the fragment chromatogram peaks of the alkadiene and the alkene molecule ions crossed (Jungwirth et al. 2021, shown for fragment ion chromatograms of other compounds in Figure S1). As the 9- and 7-alkenes of the same chain length elute directly after each other, both alkenes of the respective chain length were then integrated together, starting at the endpoint of the alkadiene-integration. This resulted in six adjusted compound class peaks: (1) 6,9-tricosadiene (C23:2); (2) 9- & 7-tricosene (C23:1); (3) 11-methyl, 9-methyl & 7-methyltricosane (MeC23); (4) 6,9-pentacosadiene (C25:2); (5) 9- & 7-pentacosene (C25:1); (6) 13-methyl, 11-methyl, 9-methyl & 7-methylpentacosane (MeC25). The integrated areas of the six adjusted compound class peaks for each chromatogram were then standardized by dividing the area of each compound class peak by the total area of the six peaks. Calculation of a Bray-Curtis dissimilarity matrix and non-metric multidimensional scaling (nMDS), and a subsequent analysis of similarity (*ANOSIM*) of the resulting groups were conducted using Primer v6 (Primer-E, Auckland, New Zealand; Clarke 1993; Clarke and Gorley 2006).

To obtain delimitation characteristics for the relative amounts of the six compound class peaks, they were analysed separately for the two chain lengths (backbone of 23 or 25 carbon atoms). The standardized amounts were re-calculated for the three compound class peaks of each chain length. For both compound chain lengths, the relative values of each compound class in each of the groups resulting from the nMDS were compared by Kruskal-Wallis tests followed by Bonferroni-Holm corrected Mann-Whitney U tests, and plotted in boxplots using R v.3.6.3 (R Core Team, 2020).

From the first generation of offspring (F1 of one to two F0 females per host obtained from the carcass traps), two females (in 19 cases three or four) were analysed by GC/MS after having had the opportunity to mate and oviposit. The chemotype identity of the F1 daughters (total n = 558) was established by visual inspection of the chromatograms based on the previously obtained chemotype delimitation characteristics. They were then compared to the respective mothers’ chemotype (total n = 262; 148 thereof were females included in the first analysis, the chemotypes of the remaining F0 females were assigned by visual inspection of the chromatograms).

### Extracts and Compound Analysis – In-depth Analyses of Chemotypes

To fully characterize the female polymorphism and to investigate whether males might show a similar differentiation in their CHC profiles, more detailed chemical analyses were conducted. Wasps for these analyses originated from chemotype-pure matrilines after a minimum of 15 generations, bred using final instar larvae of *Lucilia caesar* and *Calliphora vomitoria* as hosts (see Jungwirth et al. 2021). For each chemotype, three matrilines originating from each of the three carcass traps (n = 9 matrilines per chemotype) were selected. Always one male and one female pupa (siblings) were dissected from the same host (in all cases *L. caesar*) 1–2 days prior to eclosion from the pupal exuvia. Wasps were placed singly into 1.5 ml reaction tubes and kept at 25 °C until aged 24 to 48 h after their eclosion from the pupal exuvia. Thereafter, they were killed by freezing at -22 °C before extraction (see above, only here using 20 µl of dichloromethane with 10ng/µl of 1-eicosene as internal standard for extractions) and chemical analysis. The peaks of the resulting chromatograms were integrated manually, separating overlapping compounds where possible based on fragment ion chromatograms (Table S2). Relative peak areas were calculated for each sample by dividing the single compound areas by the respective total CHC area. Peaks comprised of two or more compounds that could not be distinguished using extracted fragment ion chromatograms due to low compound abundances for at least one of the compounds and/or similar mass spectra with low relative amounts of the diagnostic ions were treated as a single peak. Peaks with an average abundance of less than 0.1% in all sample groups were excluded from further analyses, and the relative areas were recalculated based on the total amount of the remaining CHCs. For multivariate analysis, values were square-root transformed (mitigating the influence of large peaks) before calculating a Bray-Curtis dissimilarity matrix followed by nMDS and ANOSIM as described before. We additionally calculated similarity percentages (SIMPER) using Primer v6 (Primer-E, Auckland, New Zealand; Clarke 1993; Clarke and Gorley 2006). To evaluate quantitative differences in the overall chain length composition of compounds, the proportions of hydrocarbons of the three most abundant chain lengths (23, 25, and 27 carbon atoms) were calculated by summing up the relative amounts of all hydrocarbons of the respective chain length for each sex and chemotype. Comparisons were conducted separately for males and females for each of the three chain lengths, using Kruskal-Wallis tests followed by pairwise Bonferroni-Holm corrected Mann-Whitney U tests in R v.3.6.3 (R Core Team, 2020).

### Coupled Gas Chromatography/Mass Spectrometry (GC/MS)

Samples were analysed on a Shimadzu GC/MS QP2010Plus (Shimadzu Corporation, Kyoto, Japan). For the first characterization of the chemotypes and the comparisons between F0 females and their female offspring, the GC oven was fitted with a 30 m BPX-5 column (0.25 mm diameter, 0.25 μm film thickness). One microliter per sample was injected in splitless mode at an injection temperature of 300 °C. The oven temperature was raised from 150 to 220 °C at a rate of 20 °C per minute, and thereafter to 300 °C at a rate of 8 °C per minute. In the following minute, the temperature was raised to 310 °C, at which it was held for 5 min. For the more in-depth analyses of the samples of females and males, the oven was fitted with a 60 m BPX-5 column (0.25 mm diameter, 0.25 μm film thickness). Here, the temperature program started at 150 °C and was raised to 190 °C at a rate of 8 °C per minute. Thereafter, the heating rate was 2 °C per minute until the temperature reached 310 °C, and the final temperature was held for 30 min. Of each sample, 1.5 µl were injected in splitless mode at an injection temperature of 300 °C. For both analysis settings, Helium served as carrier gas with a flow rate of 1.73 ml per minute.

## Results

**Fig. 1:**
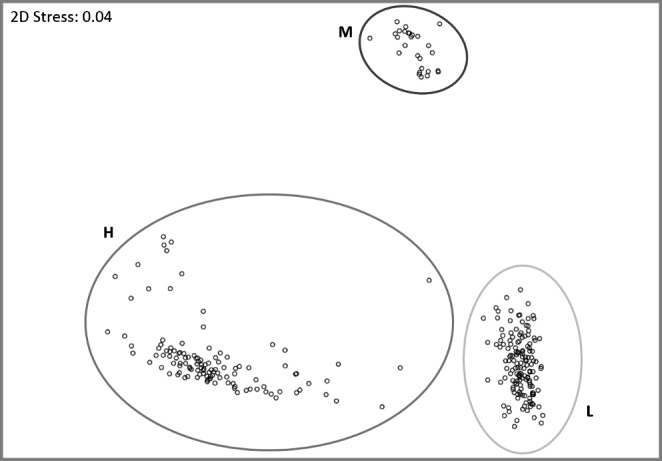
Non-metric multidimensional scaling plot based on six focal cuticular hydrocarbon (CHC) peaks from 290 females of *T. zealandicus*. The six compound class peaks covered a total of thirteen compounds belonging to three different compound classes (alkadienes, alkenes, and monomethyl-branched alkanes) of two different chain lengths (23 and 25 carbon atoms) and were adjusted to comprise only compounds of the same compound class and chain length each. See text for clarification of group naming

**Fig. 2:**
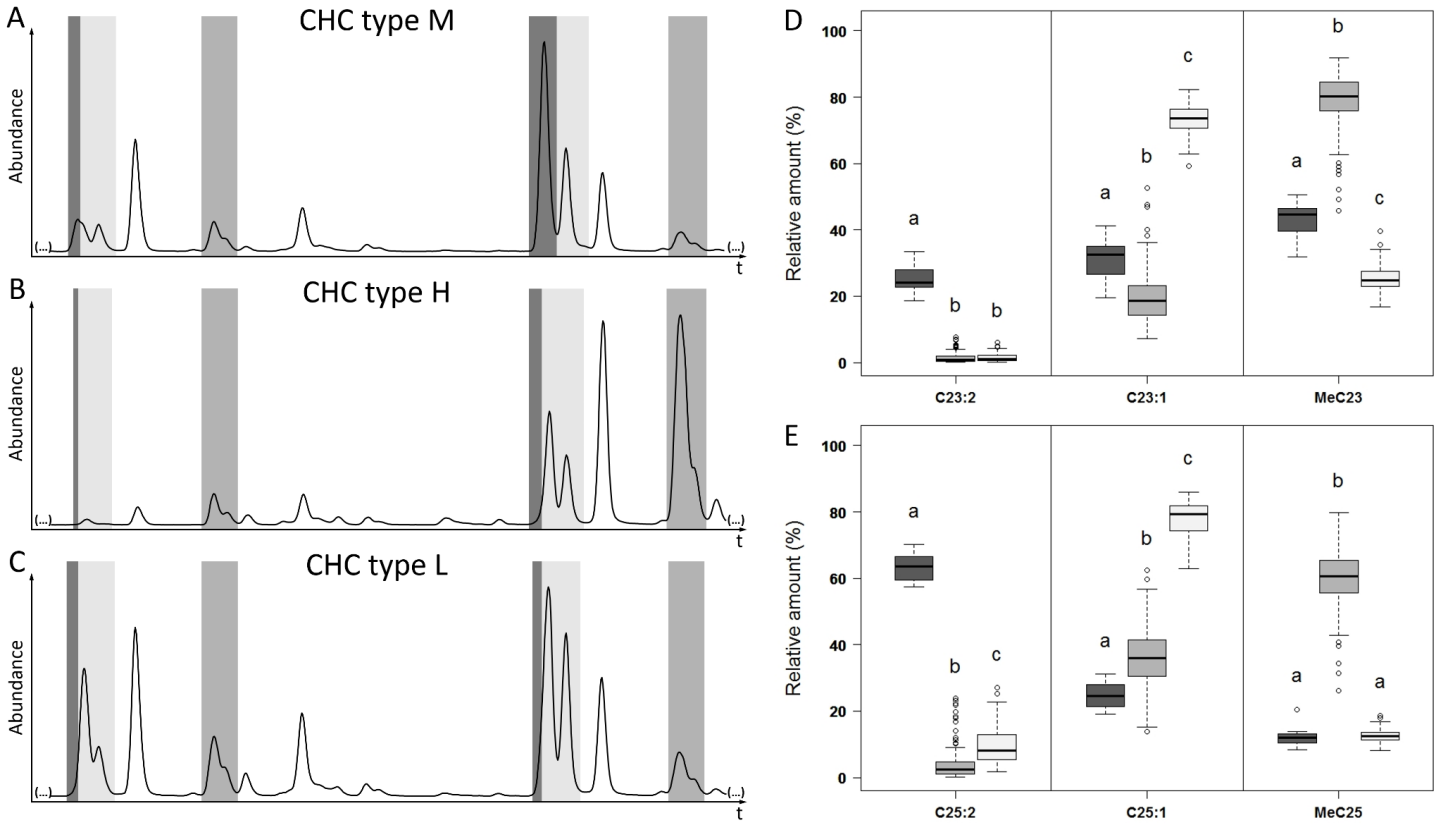
Representative partial chromatograms of the three female *T. zealandicus* chemotypes (A-C) and the respective relative amounts of the six focal cuticular hydrocarbon (CHC) compound class peaks (D, E). Each chemotype was dominated by CHCs of one compound class: (A) type M by alkadienes, (B) type H by methylbranched alkanes, (C) type L by alkenes. Integration of CHCs was adjusted to cover only one compound class and chain length each: (1) C23:2 (6,9-tricosadiene); (2) C23:1 (9- and 7-tricosene); (3) MeC23 (11-methyl, 9-methyl and 7-methyl tricosane); (4) C25:2 (6,9-pentacosadiene); (5) C25:1 (9- and 7-pentacosene); (6) MeC25 (13-methyl, 11-methyl, 9-methyl and 7-methyl pentacosane). Relative amounts of the focal compounds were calculated separately for the chain lengths of (D) 23 carbon atoms, (E) 25 carbon atoms. Boxplots depict median, second and third quartile, whiskers encompass the first and fourth quartile. Outliers (> 1.5x box height) depicted by open circles. Comparison of relative amounts of each compound class and chain length for the three female chemotypes by Kruskal-Wallis tests followed by Bonferroni-Holm corrected Mann-Whitney U tests. Different letters above the boxes indicate significant differences between the chemotypes

Based on the six selected CHC compound class peaks, the 290 samples clustered in three distinct groups (*ANOSIM*: overall R = 0.998, P < 0.001, pairwise comparisons: R ≥ 0.997, P < 0.001, Fig. 1; cluster designation M, H, and L explained below). In total, the distinct cluster M was constituted of 27 samples (9.3%), 117 (40.3%) were positioned in cluster H, and cluster L consisted of the final 146 samples (50.3%, Fig. 1). The analyses of the relative amounts of the alkadienes, alkenes, and methyl-branched alkanes for each of the two focal chain lengths revealed that the three chemotypes could be delimited by the relative ratios of these compound classes. Females from the distinct cluster M were characterized by markedly higher amounts of alkadienes compared to those found in females from the other two clusters (*Bonferroni-Holm* corrected *Mann-Whitney U tests*, P < 0.001, Table S3, Fig. 2). Overall, the relative compositions of the CHC profiles of these females were similar to those of males (see Jungwirth et al. 2021), and thus this chemotype was dubbed “type M” (**M**ale-like). In contrast, females of the two remaining clusters produced CHC profiles that were easily distinguishable from male CHC profiles, as alkadienes were present in much lower relative amounts (Fig. 2). Female CHC profiles of the cluster H differed most obviously from the other two female types, being characterized by high relative amounts of the monomethylalkanes (clearly higher than the amounts present in the profiles of females from the other two clusters, *Bonferroni-Holm* corrected *Mann-Whitney U tests*, P < 0.001, Table S3, Fig. 2). The chemotype will henceforth be referred to as “type H” (**H**igh relative amounts of methylbranched CHCs). The final chemotype was designated as “type L” (**L**ow relative amounts of methylbranched CHCs), as it could be distinguished from the aforementioned chemotype H based on the lower relative amounts of monomethylalkanes, instead producing large relative amounts of alkenes (*Bonferroni-Holm* corrected *Mann-Whitney U tests*, P < 0.001, Table S3, Fig. 2).

The comparison of CHC profiles of 262 F0 females (mated with a male that had emerged from the same host) with those of two to four of their female offspring (total n = 558), revealed that while F1 females often showed the same chemotypes as their mother, this was not exclusively the case. Of 37 F0 females with chemotype M, all analysed daughters matched the maternal chemotype in 18 cases (49%). For 132 mothers of chemotype H, this was true for 79 of them (60%), and the percentage for chemotype L was even higher (67 of 93 F0 belonging to chemotype L, 72%). For the remaining F0 females of all three chemotypes, one or all of the analysed offspring exhibited a chemotype differing from that of their mother. In one case, four F1 sisters even showed two different chemotypes, both of which did not match that of their mother. Overall, F0 females of all three chemotypes were found to be able to produce daughters exhibiting either of the three chemotypes (Table S4).

**Fig. 3:**
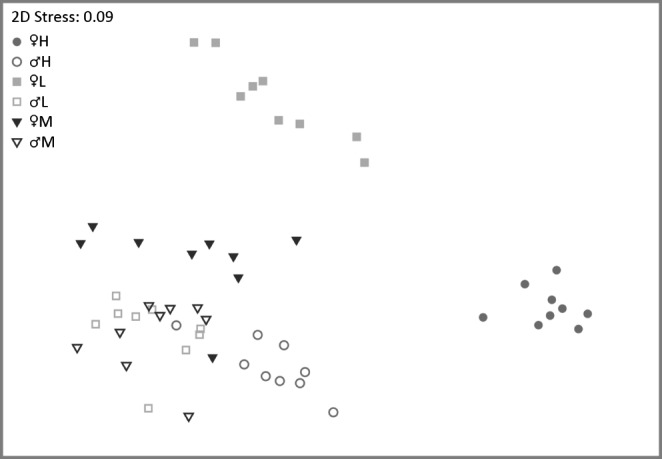
Non-metric multidimensional scaling representation of cuticular hydrocarbon (CHC) profiles of nine males (open symbols) and nine females (filled symbols) each from all three *T. zealandicus* chemotypes (type M: dark grey triangles, type H: grey circles, type L: light grey squares). All groups except for the males of types M and L were positioned in distinguishable clusters (*Analysis of Similarity*, global R = 0.809, P < 0.001, Bonferroni-Holm corrected pairwise comparisons, males M vs. males L: R = 0.021, P = 0.314; all other comparisons: R > 0.481, P < 0.001). The CHC profiles from females of type M were most similar to CHC profiles of males of all three types, while the CHC profiles from females of types H and L were positioned in clearly distinct clusters (pairwise comparisons: R-values of R = 0.845 and above)

The more detailed analyses of the full CHC profiles of both sexes included 75 CHC peaks. Eighteen of these peaks were comprised of two or more compounds that could not be separated using fragment ion chromatograms due to very similar mass spectra with diagnostic ions of low relative abundance and/or low relative amounts of at least one of the compounds (Table S5). Overall, as in the analysis including only a few selected compounds, females clearly clustered in three separate groups corresponding to their chemotype lines (*ANOSIM*: overall R = 0.807, P < 0.001; females M vs. females H: R = 1, P < 0.001; females M vs. females L: R = 0.835, P < 0.001, females H vs. females L: R = 0.992, P < 0.001; Fig. 3).

**Fig. 4:**
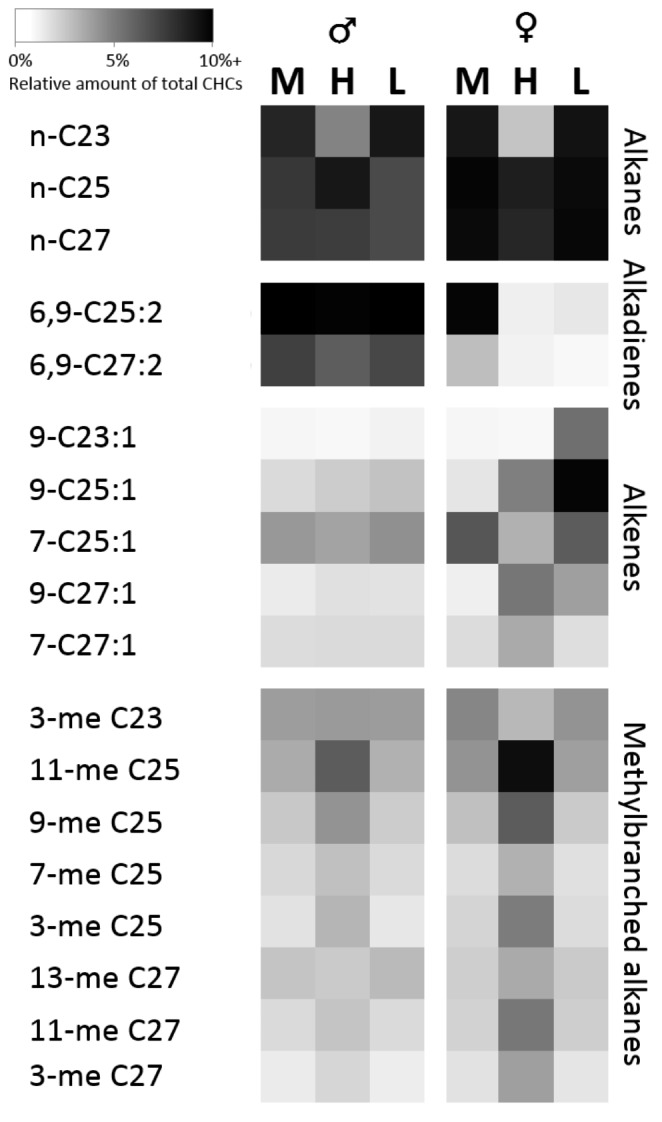
Heatmap of the relative amounts (darker for higher relative amounts) of a subset of cuticular hydrocarbons (CHCs, compound abbreviations: see text) with an average abundance of at least 2.5% in one or more of the six sample groups (males and females of the three chemotypes M, H, and L). Compounds are sorted by CHC compound class and therein by the order of elution. Heatmap-colour black used for compounds with an average amount of ≥ 10%. Relative amounts larger than 10% were found for 6,9-C25:2 in all males and females of type M, for C25 in females of type M, and for 9-C25:1 in females of type L

With the exception of one n-alkane, the first six compounds contributing the most to group dissimilarities were always alkadienes, alkenes, and methylbranched alkanes. Females of chemotypes H and M were most dissimilar in the relative abundances of C25:2, 9-heptacosene (9-C27:1), n-tricosane (n-C23), 9-pentacosene (9-C25:1), 11-methyl pentacosane (11-me C25), and 11-methylheptacosane (11-me C27; listed in order of contribution to the dissimilarity). The alkadiene and n-alkane were more abundant in females M, while the other four compounds were more abundant in females of chemotype H. In the comparison of the female chemotypes H and L, the six compounds contributing the most to the dissimilarity were 9-tricosene (9-C23:1), n-C23, 9-C25:1 (all three more abundant in females L), 11-me C25, 3-methylpentacosane (3-me C25), and 9-me C25, with the methylbranched hydrocarbons being more abundant in females H. Between females of the types L and M, differences in relative amounts were most pronounced for C25:2, 9-C25:1, 9-C23:1, 9-C27:1, 6,9-heptacosadiene (C27:2), and C23:2. The three alkenes were of higher abundance in females L, while the three alkadienes were more abundant in females M (Figure S2). Overall, females of type M clustered the closest to all males (*ANOSIM*, pairwise comparisons of females M vs. male groups: lowest R = 0.483 and largest R = 0.703; for females of H or L vs. males, lowest R = 0.945, Fig. 3). Male clusters were in closer proximity to each other than the clusters of females (Fig. 3). The CHC profiles of males whose sisters exhibited the chemotypes M and L could not be distinguished (*ANOSIM* R = 0.019, P = 0.325; Fig. 3), while brothers of H type females were positioned in a distinguishable cluster (*ANOSIM*: males H vs. males M: R = 0.639, P < 0.001; males H vs. males L: R = 0.711, P < 0.001; Fig. 3). Overall, the pattern of relative amounts for all compounds contributing a minimum average amount of 2.5% to at least one of the six groups indicated that brothers of chemotype H females differed from other males by having relatively less n-C23 and less alkadienes, but relatively more methylbranched alkanes (C25 and higher, Fig. 4). Indeed, the six compounds contributing the most to group dissimilarity were n-C23, 6,9-C25:2, 11-MeC25, 3-MeC25, 9-MeC25, and 6,9-C27:2, with the methylbranched alkanes always being of a higher relative abundance for males originating from chemotype H. The relatively lower amount of n-C23 in combination with a higher amount of methylbranched CHCs of chain length C25 and above found in these males mirrored the pattern shown by females of chemotype H, though the relative amount of methylbranched CHCs was almost always higher in the females (Fig. 4, Table S5).

**Fig. 5:**
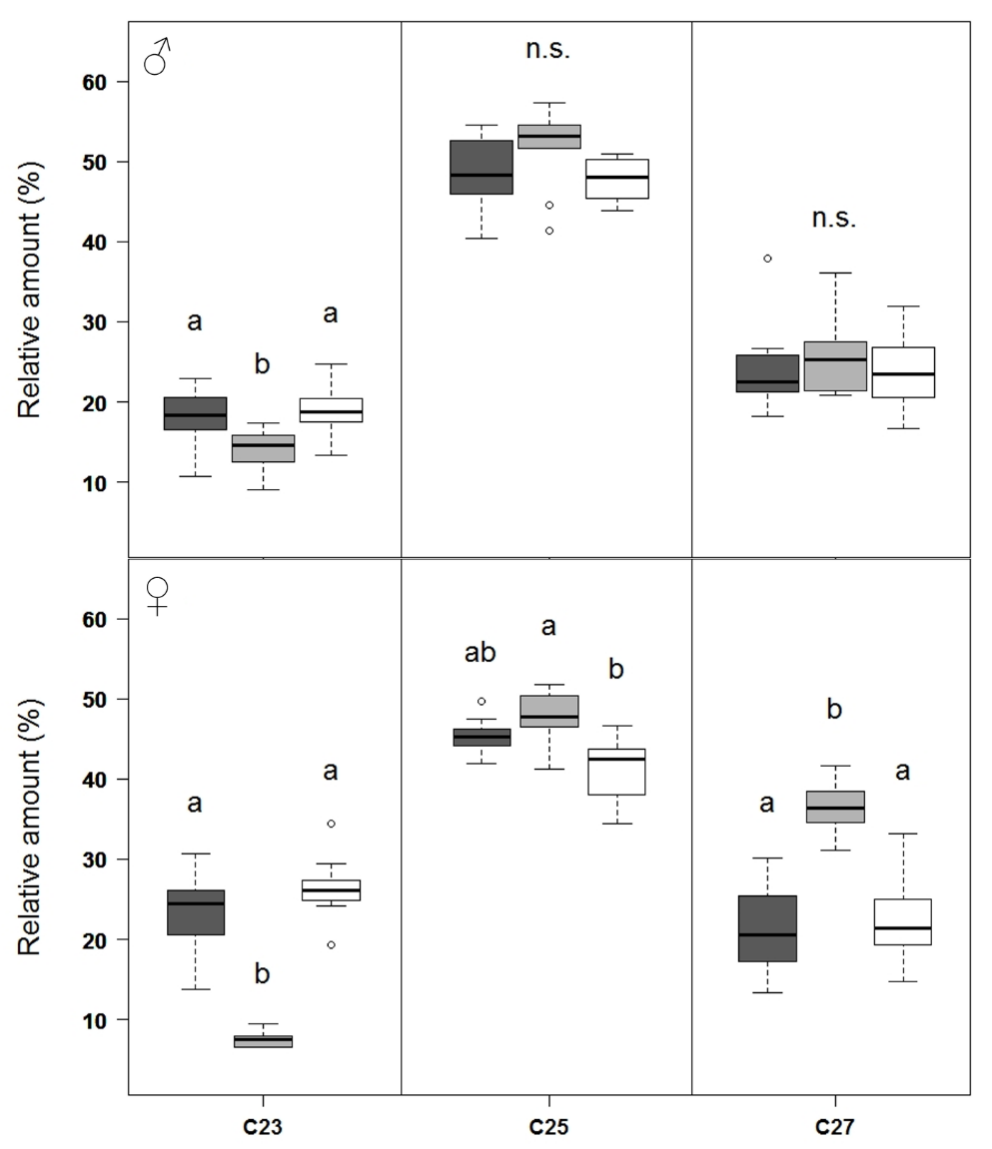
Summed relative amounts for cuticular hydrocarbons (CHCs) with carbon backbone chainlengths of 23 (C23), 25 (C25) and 27 (C27) carbon atoms. Comparison of (A) males and (B) females from the three chemotypes M (dark grey), H (grey), and L (light grey) of *T. zealandicus*. Boxplots depict median, second and third quartile, whiskers encompass the first and fourth quartile. Outliers (> 1.5x box height) depicted by open circles. Kruskal-Wallis tests followed by pairwise Bonferroni-Holm corrected Mann-Whitney U tests, significant differences between the chemotypes indicated by different letters, n.s. = not significant (for detailed results see Table S4)

In addition to the quantitative differences in hydrocarbon classes, the wasps showed differences in the relative amounts of compounds of the three dominant chain lengths. While males of all three chemotypes did not differ in the summed relative amounts of compounds with 25 or 27 carbon atoms, males belonging to chemotype H exhibited significantly less CHCs with a chain length of 23 carbons than those of the other two chemotypes (Fig. 5 A, Table S6). In females, relative amounts of CHCs with a C23 backbone were also significantly lower in H type females than in females of the other two chemotypes. Additionally, they differed in the other two chain lengths, with females of chemotype H producing significantly more C25-CHCs than females of chemotype L, and more CHCs with a chain length of 27 carbon atoms than both, females L and females M (Fig. 5B, Table S6, Figure S2). Thus, H type female CHC profiles were characterized by an overall shift of relative compound amounts towards the longer chain lengths.

## Discussion

The chemical analyses clearly showed that the wasps produce distinct intrasexual CHC phenotypes differing in the relative amounts of shared compounds. Females showed three distinct chemotypes. Each chemotype was characterized by one of three compound classes dominating in the respective profiles: alkadienes in Type M (very similar to male profiles), methylbranched alkanes in type H, and alkenes in type L. Male profiles were much more similar to each other and clustered closely, but could, nonetheless, be separated into two different chemotypes. All males produced large amounts of alkadienes, but only sons of H females produced slightly elevated relative amounts of methylbranched CHCs. This is roughly similar to *P. triangulum*, where the male CHC dimorphism mirrored the female one, though to a lesser extent (Kroiss et al. 2006; Strohm et al. 2008b; Strohm pers. comm.). However, male *T. zealandicus* of both, the M and L types, showed a CHC profile similar to that of females exhibiting the M chemotype, while these females produced overall lower relative amounts of dienes than males of any type. The CHC profiles of L males did not mirror the characteristics (elevated amounts of alkenes) of the L females.

There was no indication of cryptic species, as field-trapped F0 females of all three chemotypes produced offspring of either chemotype (in some cases even two differing chemotypes). While the CHC profile of insects has been shown to be influenced by e.g. food source (Liang and Silverman 2000; Geiselhardt et al. 2012) or temperature (Menzel et al. 2017b; Rajpurohit et al. 2021), neither of these factors are likely to be the cause of the observed chemotypes. Once in the laboratory, the wasps were kept at the same temperatures. Additionally, the wasps used for the detailed analyses of CHC profiles were all obtained from the same host species and were furthermore of the same ages and sexually mature (the wasps usually mate directly after emergence (Johnston and Tiegs 1921; Olton and Legner 1974; personal observation), and take approximately one day to emerge from the host after eclosion from the pupal exuvia (personal observation, and see Olton and Legner 1974)). Other factors potentially influencing CHC profiles, such as mating status or oviposition, can also be excluded. All three chemotypes were present among F0 females of the first analysis which were mated and had oviposited, as well as among females from the inbred matrilines of the second analysis, which were virgin and had not laid eggs. While minor influences of the above factors on the CHC profiles will still be studied in a controlled setup, the overall consistency of the chemotypes found here (together with the corresponding male and female chemotypes of M and H in inbred matrilines outlined before) indicates molecular causes for the CHC polymorphism. The mode of inheritance of within-population CHC polymorphism is, to our knowledge, unknown for either of the other described cases of CHC variation within a population (*O. spinipes*, *P. triangulum*, *E. dilemma*). In *Drosophila melanogaster*, females from different populations are characterized by producing high quantities of 7,11-heptacosadiene or the positional isomer 5,9-heptacosadiene, with none or only low relative amounts of the respective other compound. Ferveur et al. (1996) showed that crossbred F1 offspring produced both heptacosadienes in relative amounts that were intermediate to the two parent lines. The heritability of the CHC profiles in *T. zealandicus* will be elucidated in future investigations, using the now established, chemotype-pure matrilines.

One of the most interesting aspects of the three female chemotypes to be studied hereafter will be the effect on sexual communication. The CHCs of M type females elicit mating attempts from M type males despite the similarity of the profiles (Jungwirth et al. 2021). The consequences of the CHC polymorphism for mate recognition are as yet unknown. In *D. melanogaster*, both female chemotypes were observed to elicit male courtship behaviour equally well despite originating from separate populations. However, the cumulative percentage of mated females within 10 min was higher for the cosmopolitan female chemotype carrying larger amounts of 7,11-heptacosadiene (Ferveur et al. 1996, but see Savarit et al. 1999; Ferveur 2005 on the complex roles of this compound and other CHCs for courtship and mating). In contrast, in the case of within-population variation of the volatile, non-CHC, sex pheromone of *Heliothis virescens*, females producing unusually high relative amounts of saturated aldehydes were less effective in attracting males than females producing the more common sex pheromone composition (Groot et al. 2014). In a case of CHC plasticity in response to differing food sources, the mustard leaf beetle *Phaedon cochleariae* preferred females raised on the same host plant over those raised on a different host plant, with the response based on the females’ respective CHC profiles (Geiselhardt et al. 2012). Future studies will assess if and how the differing CHC profiles of *T. zealandicus* affect mate choice.

The three CHC chemotypes occur in different frequencies in the studied population. The two most common chemotypes were H and L, with randomly chosen F0 females (one per obtained host) representing 37–43% (H) and 40–62% (L) of all analysed samples for each of the three carcass traps. Chemotype M was much rarer, constituting 1–18% of the analysed F0 females. While there likely is a bias towards females better at finding and ovipositing in hosts before the carcass trap was removed from the field, it is unknown whether chemotype identity is correlated with host finding performance. Nonetheless, we believe that the results of the analyses presented here are, overall, a reasonable representation of chemotype frequencies for this population. A total of 162 dead females (that may have died after parasitizing fly larvae) had been found in the three carcass traps. Additionally, further females might have oviposited, but not died in the traps. Thus, the females emerging from the 290 obtained hosts that were analysed here are unlikely to be the offspring of only very few wasps, and therefore presumably reflect naturally occurring chemotype frequencies from the sampled population. In *O. spinipes*, where female CHC chemotypes differ to a large extent by being composed of different compounds (Wurdack et al. 2015), a large-scale study of hundreds of individuals from across the European range of this species revealed that chemotype frequencies differed markedly between populations (Moris et al. 2021). It will be interesting to investigate whether chemotype frequencies differ between populations in *T. zealandicus*, and whether climate or season are correlating factors. Climatic factors have been found to correlate with the relative abundances of specific CHC classes in the cuticular lipid profiles of ants (Menzel et al. 2017a), and experimental manipulation of/acclimatization to temperature and humidity were found to lead to quantitative changes in CHC profiles of ants and fruit flies (Menzel et al. 2017b; Sprenger et al. 2018; Rajpurohit et al. 2021). The differing CHC chemotypes of female *T. zealandicus* could be advantageous under different climatic regimes. For example, H-type females, with the slight overall shift towards longer chain lengths and elevated amounts of methylbranched compounds rather than alkadienes or alkenes that generally have lower melting temperatures than methyl-branched alkanes (Gibbs and Pomonis 1995), might perform better under warmer, drier conditions. If this were the case, it would be expected that the relative chemotype frequency in a given population would reflect the prevalent climatic conditions.

This study is to our knowledge the first to show three clearly distinct CHC chemotypes in females as well as two in the overall more similar profiles of males within a single population of a solitary insect. It lays the foundation for our future investigations of the chemo-ecological consequences of variability in CHC profiles that in many species serve a dual function as desiccation barrier and for communication.

## Electronic Supplementary Material

Below is the link to the electronic supplementary material.

Supplementary Material 1
