# Supplementary material for: Cuticular Hydrocarbon Polymorphism in a Parasitoid Wasp
Source: J Chem Ecol. 2023 Jan 27;49(1-2):36–45. doi: 10.1007/s10886-022-01401-2 (PMC9941234; doi:10.1007/s10886-022-01401-2)
Supplement: Supplementary file 1 — Supplementary Material 1 [file 10886_2022_1401_MOESM1_ESM.pdf]

Cuticular hydrocarbon polymorphism in a parasitoid wasp

Tamara Pokorny* and Joachim Ruther

Institute of Zoology, University of Regensburg, Regensburg, Germany
*corresponding author: tamara.pokorny@biologie.uni-regensburg.de

Electronic Supplementary Material

6 Tables, 2 Figures

Table S1: Molecule ions and display factors of ion chromatograms used to separate overlapping alkadienes and alkenes in extract chromatograms of F0 females of *T. zealandicus*. As the molecule ion fragments of 6,9-pentacosadiene (6,9-C25:2) and 9-pentacosene (9-C25:1) constituted roughly double the proportional amount to the respective mass spectra than those of 6,9-tricosadiene (6,9-C23:2) and 9-tricosene (9-C23:1), the display factor for the ion chromatograms was halved.

Compound	Molecule ion	Display factor for ion chromatogram
6,9-C23:2	320	100
9-C23:1	322	175
6,9-C25:2	348	50
9-C25	350	87.5

Table S2: Separation parameters for overlapping peaks in the detailed analyses of chemotype pure matrilines. Separable compounds listed with respective characteristic fragment ions and display factors (evaluated from representative mass spectra; adjusted so that similar amounts of compounds yield similar heights of fragment ion chromatogram peaks). Compound abbreviations: 'Cn' = chain length n; 'x-me'/'x-C' = methyl-branch/double-bond at position x; ':1' = alkene; ':2' = alkadiene; 'tr' = trace. Overlapping peaks indicated by boxes surrounding the group of compounds contained therein.

Compound	Fragment ion	Display factor
11-me C23	168	78.6
9-me C23	140	39.4
6,9-C25:2 & tr: 3-me C24	110	28.6
9-C25:1	111	23.3
x,x-C25:2	110	28.6
7-C25:1	111	23.3
y,y-C25:2	110	28.6
z,z-C25:2	110	28.6
13-me C25	196	57.3
11-me C25	168	78.6
9-me C25	140	39.4
11,x-dime C25	168	78.6
9,13-dime C25	140	39.4
3-me C25	111	23.3
6,9-C26:2	110	28.6
6,9-C27:2 & tr: 3-me C26	110	28.6
9-C27:1	111	23.3
x,x-C27:2	110	28.6
7-C27:1	111	23.3
13-me C27	196	57.3
11-me C27	168	78.6
9-me C27	140	39.4
11,15-dime	168	78.6
9,13-dime & 9,15-dime C27	140	39.4
6,9-C29:2	110	28.6
9-C29:1	111	23.3
15-me C29	224	57.3
13-me C29	196	57.3
11-me C29	168	78.6
9-me C29	140	39.4

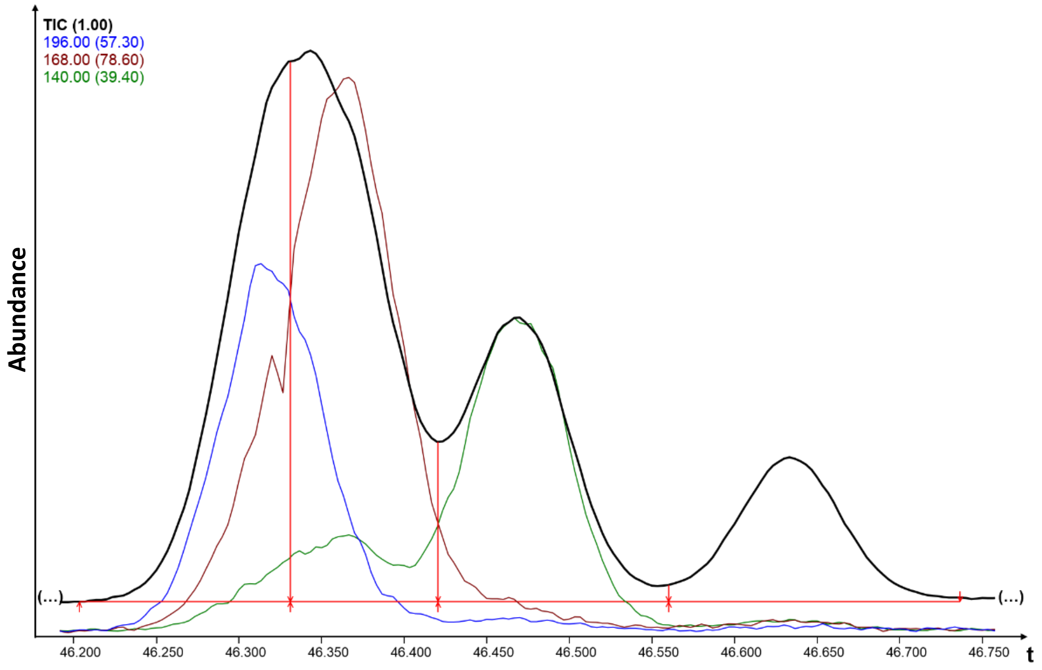

Figure S1: Example of peak separation based on fragment ion chromatograms (here: 13-me C27, 11-me C27, and 9-me C27; 7-me C27 elutes last, as a separate peak). The total ion current chromatogram (TIC, black) is displayed together with the relevant fragment ion chromatograms (blue, brown, and green; display factors listed in brackets behind the respective fragment ions). Peaks are split where the two relevant fragment ion chromatograms cross, the red arrows indicate the split positions. Peak areas (integration borders: TIC between two split positions and the red baseline) can then be calculated separately for each of the resulting peaks.

Table S3: Results of overall and pairwise comparisons of the relative amounts of alkadienes (:2), alkenes (:1), and methylbranched alkanes (me-) for chainlengths of 23 (C23) and 25 (C25) carbon atoms. Kruskal-Wallis tests followed by Bonferroni-Holm corrected pairwise Mann-Whitney U tests.

Comparison C23	χ^2	W	P-value	Comparison C25	χ^2	W	P-value
6,9-C23:2	76.338; df = 2		<2.2E-16	6,9-C25:2	151.24; df = 2		<2.2E-16
pairwise: females M - females H		3159	1.3E-15 [a]	pairwise: females M - females H		3159	6.4E-16 [a]
pairwise: females M - females L		3942	6.6E-16 [a]	pairwise: females M - females L		3942	4.4E-16 [a]
pairwise: females H - females L		7345	0.051 [a]	pairwise: females H - females L		2571	6.6E-16 [a]
9-C23:1 & 7-C23:1	225.76; df = 2		<2.2E-16	9-C25:1 & 7-C25:1	225.73; df = 2		<2.2E-16
pairwise: females M - females H		2755	1.8E-09 [a]	pairwise: females M - females H		402	1.70E-09 [a]
pairwise: females M - females L		0	4.4E-16 [a]	pairwise: females M - females L		0	4.4E-16 [a]
pairwise: females H - females L		0	6.6E-16 [a]	pairwise: females H - females L		0	6.6E-16 [a]
11-me, 9-me & 7-me C23	232.43; df = 2		<2.2E-16	13-me, 11-me, 9-me & 7-me C25	209.81; df = 2		<2.2E-16
pairwise: females M - females H		9	9.3E-16 [a]	pairwise: females M - females H		0	1.3E-15 [a]
pairwise: females M - females L		3930	5.1E-16 [a]	pairwise: females M - females L		1544	0.074 [a]
pairwise: females H - females L		17082	6.6E-16 [a]	pairwise: females H - females L		17082	6.6E-16 [a]

[a] Bonferroni-Holm corrected P-values

Table S4: Chemotype identities of two to four female F1 offspring (total n = 558) compared to the respective mother's chemotype (total n = 262). For each F0 female, two to four F1 females were analysed. Listed are the number of cases in which the respective offspring chemotypes were observed.

Chemotype F0	All F1 same chemotype as F0	All F1 other chemotype than F0	F1 mixed chemotypes
M (n = 37)	18	H: 5<br>L: 11	M & L: 2<br>H & L: 1
H (n = 132)	79	M: 1<br>L: 13	M & H: 4<br>H & L: 35
L (n = 93)	67	M: 3<br>H: 17	M & L: 4<br>H & L: 2

Table S5: Average relative amounts of the cuticular hydrocarbons (CHCs, ± SD) of *T. zealandicus* contributing more than 0.1% to the CHC profiles of at least one of the six sample groups (average across n = 9 samples of each males and females of the three chemotypes M, H, and L). Compounds that could not be separated using fragment ion chromatograms were integrated together in one peak. Compound abbreviations as in Table S2.

Compound	LRI	♂ M	♂ H	♂ L	♀ M	♀ H	♀ L
n-C21	2100	0.47 ± 0.11	0.21 ± 0.08	0.56 ± 0.17	0.58 ± 0.28	0.02 ± 0.01	0.67 ± 0.25
n-C22	2200	0.22 ± 0.04	0.14 ± 0.04	0.22 ± 0.05	0.23 ± 0.05	0.09 ± 0.03	0.22 ± 0.05
6,9-C23:2	2272	0.60 ± 0.19	0.35 ± 0.09	0.60 ± 0.29	0.93 ± 0.72	0.01 ± 0.03	0.06 ± 0.04
9-C23:1	2276	0.25 ± 0.09	0.20 ± 0.08	0.44 ± 0.20	0.30 ± 0.17	0.17 ± 0.07	5.60 ± 2.87
7-C23:1	2282	0.42 ± 0.14	0.17 ± 0.07	0.50 ± 0.20	1.13 ± 0.66	0.03 ± 0.01	1.17 ± 0.52
n-C23	2300	8.69 ± 1.65	4.77 ± 1.64	9.22 ± 1.66	9.24 ± 1.64	2.22 ± 0.22	9.44 ± 1.08
11-me C23	2334	1.27 ± 0.44	1.43 ± 0.47	1.20 ± 0.41	2.31 ± 0.91	0.95 ± 0.18	1.89 ± 0.48
9-me C23	2336	1.16 ± 0.42	0.97 ± 0.32	1.17 ± 0.39	1.76 ± 0.55	0.13 ± 0.05	1.45 ± 0.38
7-me C23	2340	0.80 ± 0.30	0.97 ± 0.24	0.77 ± 0.21	1.26 ± 0.38	0.39 ± 0.10	1.08 ± 0.21

5-me C23	2349	0.38 ± 0.08	0.35 ± 0.06	0.41 ± 0.13	0.58 ± 0.27	0.24 ± 0.05	0.67 ± 0.30
9,13-dime C23	2364	0.07 ± 0.03	0.10 ± 0.03	0.05 ± 0.02	0.14 ± 0.06	0.11 ± 0.05	0.08 ± 0.02
7,11-dime C23	2368	0.18 ± 0.06	0.22 ± 0.06	0.15 ± 0.04	0.27 ± 0.10	0.09 ± 0.02	0.17 ± 0.04
3-me C23	2372	3.79 ± 0.45	3.89 ± 0.48	3.85 ± 0.54	4.69 ± 0.60	2.69 ± 0.52	4.17 ± 0.66
9-C24:1	2375	0.05 ± 0.01	0.06 ± 0.01	0.06 ± 0.01	0.06 ± 0.02	0.05 ± 0.02	0.22 ± 0.08
5,9-dime C23 & 5,11-dime C23 & x-C24:1	2379	0.24 ± 0.05	0.27 ± 0.05	0.22 ± 0.04	0.30 ± 0.08	0.24 ± 0.08	0.22 ± 0.04
5,13-dime C23 & 7-C24:1	2383	0.09 ± 0.01	0.09 ± 0.02	0.08 ± 0.02	0.14 ± 0.06	0.06 ± 0.03	0.13 ± 0.03
5,17-dime C23	2388	0.06 ± 0.03	0.05 ± 0.02	0.05 ± 0.03	0.16 ± 0.16	0.14 ± 0.12	0.15 ± 0.10
n-C24	2400	0.46 ± 0.06	0.44 ± 0.07	0.45 ± 0.06	0.64 ± 0.10	0.31 ± 0.04	0.62 ± 0.13
3,11-dime C23 & 3,9-dime C23 & 3,7-dime C23	2406	0.20 ± 0.04	0.26 ± 0.05	0.18 ± 0.04	0.32 ± 0.06	0.12 ± 0.04	0.23 ± 0.06
Monomethyls C24 & 3,5-dime C23	2433	0.29 ± 0.07	0.51 ± 0.12	0.27 ± 0.06	0.33 ± 0.08	0.47 ± 0.08	0.30 ± 0.10
4-me C24	2456	0.22 ± 0.04	0.26 ± 0.05	0.23 ± 0.06	0.31 ± 0.06	0.36 ± 0.09	0.27 ± 0.05
6,9-C25:2	2474	24.33 ± 4.83	16.73 ± 3.17	23.8 ± 2.95	13.88 ± 4.53	0.53 ± 0.72	0.86 ± 0.55
9-C25:1	2477	1.35 ± 0.33	1.95 ± 0.67	2.35 ± 0.73	0.99 ± 0.44	5.01 ± 0.96	13.95 ± 5.02
x,x-C25:2	2480	0.73 ± 0.20	0.51 ± 0.11	0.72 ± 0.09	0.33 ± 0.10	0.02 ± 0.02	0.02 ± 0.01
7-C25:1	2484	3.98 ± 0.48	3.57 ± 0.72	4.28 ± 0.87	6.65 ± 1.70	2.96 ± 0.97	6.37 ± 2.29
y,y-C25:2	2487	0.14 ± 0.03	0.11 ± 0.03	0.13 ± 0.02	0.17 ± 0.04	0.09 ± 0.02	0.10 ± 0.03
z,z-C25:2	2491	0.11 ± 0.02	0.07 ± 0.03	0.09 ± 0.03	0.20 ± 0.09	0.02 ± 0.01	0.03 ± 0.01
n-C25	2500	7.93 ± 1.16	9.24 ± 1.48	7.14 ± 1.03	11.15 ± 2.36	8.96 ± 1.19	9.78 ± 2.55
13-me C25	2531	0.56 ± 0.14	0.81 ± 0.22	0.59 ± 0.25	0.41 ± 0.18	0.92 ± 0.30	0.40 ± 0.14
11-me C25	2533	3.21 ± 0.54	6.38 ± 1.81	3.00 ± 0.69	4.17 ± 1.12	9.70 ± 1.81	3.70 ± 1.31
9-me C25	2535	2.11 ± 0.27	4.14 ± 0.92	1.95 ± 0.29	2.40 ± 0.50	6.40 ± 0.51	1.99 ± 0.41
7-me C25	2540	1.41 ± 0.19	2.39 ± 0.37	1.35 ± 0.26	1.26 ± 0.24	3.03 ± 0.30	1.10 ± 0.18
5-me C25	2549	0.52 ± 0.13	0.60 ± 0.12	0.55 ± 0.18	0.63 ± 0.17	1.22 ± 0.30	0.62 ± 0.21
11,x-dime C25	2560	0.03 ± 0.01	0.06 ± 0.03	0.02 ± 0.01	0.06 ± 0.02	0.16 ± 0.03	0.04 ± 0.02
9,13-dime C25	2561	0.21 ± 0.03	0.35 ± 0.09	0.20 ± 0.05	0.26 ± 0.06	0.50 ± 0.10	0.19 ± 0.05
7,x-dime C25 (7,15- 7,11- 7, 13-)	2567	0.36 ± 0.06	0.79 ± 0.25	0.32 ± 0.08	0.43 ± 0.13	1.03 ± 0.17	0.29 ± 0.07
3-me C25	2573	1.00 ± 0.23	2.83 ± 0.66	0.88 ± 0.18	1.63 ± 0.33	5.09 ± 0.55	1.25 ± 0.28
6,9-C26:2	2575	0.33 ± 0.13	0.24 ± 0.09	0.34 ± 0.09	0.08 ± 0.05	0.01 ± 0.01	0.01 ± 0.01
x-C26:1 & 5,x-dime C25 (5,11- 5,15- 5,17-)	2578	0.38 ± 0.05	0.65 ± 0.11	0.41 ± 0.09	0.41 ± 0.10	1.07 ± 0.22	0.43 ± 0.13
n-C26	2600	0.41 ± 0.13	0.51 ± 0.08	0.39 ± 0.10	0.58 ± 0.17	0.98 ± 0.13	0.63 ± 0.33
3,9-dime C25 & 3,7-dime C25	2605	0.22 ± 0.03	0.50 ± 0.11	0.20 ± 0.03	0.26 ± 0.08	0.75 ± 0.15	0.21 ± 0.05
Monomethyls C26 & 3,5-dime C23	2631	0.26 ± 0.07	0.43 ± 0.16	0.29 ± 0.10	0.26 ± 0.05	0.84 ± 0.11	0.27 ± 0.09
6,9-C27:2	2676	7.50 ± 3.22	6.31 ± 2.64	7.24 ± 2.51	2.50 ± 1.62	0.39 ± 0.32	0.20 ± 0.15
9-C27:1	2677	0.68 ± 0.27	1.10 ± 0.38	1.07 ± 0.28	0.54 ± 0.39	5.39 ± 1.22	3.71 ± 1.43
x,x-C27:2	2681	0.15 ± 0.08	0.11 ± 0.05	0.12 ± 0.06	0.05 ± 0.05	0.01 ± 0.01	0.01 ± 0.01
7-C27:1	2685	1.30 ± 0.68	1.37 ± 0.43	1.37 ± 0.60	1.28 ± 0.71	3.24 ± 0.77	1.22 ± 0.52
n-C27	2700	7.76 ± 2.67	7.67 ± 1.92	7.08 ± 1.64	9.80 ± 3.15	8.61 ± 1.21	9.92 ± 4.27
13-me C27	2730	2.24 ± 0.45	1.99 ± 0.52	2.60 ± 1.13	1.80 ± 0.48	3.31 ± 0.78	1.99 ± 0.62
11-me C27	2731	1.32 ± 0.29	2.25 ± 0.65	1.34 ± 0.42	1.72 ± 0.62	5.31 ± 1.14	1.82 ± 0.87
9-me C27	2734	0.77 ± 0.17	1.28 ± 0.30	0.89 ± 0.42	0.71 ± 0.20	2.22 ± 0.41	0.80 ± 0.29
7-me C27	2739	0.62 ± 0.15	0.70 ± 0.17	0.71 ± 0.35	0.41 ± 0.13	1.01 ± 0.20	0.46 ± 0.14
5-me C27	2748	0.08 ± 0.05	0.09 ± 0.06	0.08 ± 0.02	0.10 ± 0.04	0.13 ± 0.04	0.09 ± 0.05
11,15-dime C27	2757	0.04 ± 0.02	0.05 ± 0.02	0.04 ± 0.01	0.07 ± 0.03	0.12 ± 0.02	0.06 ± 0.02
9,13-dime C27 & 9,15-dime C27	2759	0.14 ± 0.06	0.20 ± 0.04	0.14 ± 0.05	0.18 ± 0.06	0.37 ± 0.10	0.15 ± 0.05
7,x-dime C27 (7,15- 7,11-)	2765	0.25 ± 0.06	0.57 ± 0.20	0.24 ± 0.08	0.28 ± 0.12	1.31 ± 0.33	0.24 ± 0.08

3-me C27	2773	0.69 ± 0.31	1.54 ± 0.53	0.64 ± 0.16	1.05 ± 0.43	3.71 ± 0.69	0.95 ± 0.42
x-C28:1 & 5,x-dime C27 (5,11- 5,15- 5,17-)	2777	0.12 ± 0.06	0.16 ± 0.06	0.11 ± 0.02	0.15 ± 0.06	0.31 ± 0.12	0.16 ± 0.05
n-C28 & 3,x-dime C27 (3,15- 3,13- 3,11- 3,9- 3,7-)	2801	0.48 ± 0.17	0.63 ± 0.21	0.45 ± 0.09	0.56 ± 0.19	1.01 ± 0.28	0.53 ± 0.20
Monomethlys C28 (14- to 10-)	2830	0.19 ± 0.10	0.23 ± 0.11	0.21 ± 0.05	0.21 ± 0.06	0.40 ± 0.10	0.22 ± 0.08
6,9-C29:2	2878	0.11 ± 0.08	0.14 ± 0.14	0.10 ± 0.04	0.09 ± 0.07	0.03 ± 0.04	0.04 ± 0.02
9-C29:1	2879	0.10 ± 0.06	0.12 ± 0.08	0.13 ± 0.05	0.12 ± 0.08	0.62 ± 0.40	0.29 ± 0.14
n-C29	2900	1.55 ± 0.77	0.96 ± 0.60	1.47 ± 0.49	1.57 ± 0.69	0.58 ± 0.17	1.36 ± 0.31
15-me C29	2928	0.13 ± 0.08	0.06 ± 0.04	0.23 ± 0.12	0.09 ± 0.06	0.02 ± 0.02	0.09 ± 0.05
13-me C29	2929	0.29 ± 0.11	0.14 ± 0.06	0.25 ± 0.06	0.29 ± 0.10	0.20 ± 0.05	0.30 ± 0.11
11-me C29	2930	0.21 ± 0.10	0.38 ± 0.11	0.29 ± 0.14	0.31 ± 0.09	0.86 ± 0.19	0.42 ± 0.24
9-me C29	2934	0.28 ± 0.09	0.35 ± 0.12	0.36 ± 0.18	0.25 ± 0.08	0.39 ± 0.09	0.30 ± 0.11
Dimethyls C29	2963	0.08 ± 0.04	0.09 ± 0.04	0.09 ± 0.03	0.12 ± 0.05	0.12 ± 0.03	0.11 ± 0.06
3-me C29	2974	0.27 ± 0.13	0.33 ± 0.15	0.26 ± 0.07	0.38 ± 0.14	0.54 ± 0.22	0.36 ± 0.13
(C30 & dimethyls C29)	3000	0.14 ± 0.07	0.13 ± 0.08	0.13 ± 0.06	0.18 ± 0.10	0.20 ± 0.05	0.15 ± 0.04
11-me C31 / monomethyls C31	3131	0.18 ± 0.05	0.20 ± 0.11	0.17 ± 0.09	0.20 ± 0.11	0.19 ± 0.07	0.18 ± 0.09
3-me C31	3174	0.10 ± 0.06	0.09 ± 0.06	0.10 ± 0.04	0.15 ± 0.05	0.10 ± 0.03	0.14 ± 0.06
11-me C35 / monomethyls C35	3526	0.32 ± 0.11	0.26 ± 0.09	0.35 ± 0.17	0.25 ± 0.04	0.19 ± 0.09	0.25 ± 0.12
11-me C37	3726	1.79 ± 0.45	1.30 ± 0.65	1.62 ± 0.50	1.77 ± 0.38	0.40 ± 0.14	1.72 ± 0.97
Dimethyl(s) C37	3756	0.13 ± 0.05	0.15 ± 0.10	0.11 ± 0.04	0.15 ± 0.06	0.09 ± 0.05	0.13 ± 0.06
11-me C39	3926 [b]	0.48 ± 0.15	0.53 ± 0.25	0.33 ± 0.15	0.78 ± 0.22	0.49 ± 0.22	0.59 ± 0.25

LRI = Linear retention index

x, y, z = Unknown positions of double bonds or methylbranches

() = Tentative compound identifications

[b] RI estimated; accurate calculation of RI not possible: n-C40 of alkane-standard did not elute during run-time

Table S6: Comparisons of summed average amounts of CHCs with the three dominant chain lengths of 23, 25, and 27 carbon atoms for male and female *T. zealandicus* of all three chemotypes. Comparisons were conducted using Kruskal-Wallis tests followed by Bonferroni-Holm corrected pairwise Mann-Whitney U tests.

**Comparison males**	**χ^2**	**W**	**P-value**	**Comparison females**	**χ^2**	**W**	**P-value**
C23 CHCs	10.296; df = 2		5.8e-3	C23 CHCs	17.824; df = 2		1.3e-4
pairwise: males M - males H		69	0.021 [a]	pairwise: females M - females H		81	8.2e-5 [a]
pairwise: males M - males L		37	0.79 [a]	pairwise: females M - females L		29	0.34 [a]
pairwise: males H - males L		7	0.0056 [a]	pairwise: females H - females L		0	1.2e-4 [a]
C25 CHCs	4.1058; df = 2		0.13	C25 CHCs	8.9136; df = 2		1.2e-2
				pairwise: females M - females H		22	0.11 [a]
				pairwise: females M - females L		65	0.063 [a]
				pairwise: females H - females L		69	0.032 [a]
C27 CHCs	0.98413; df = 2		0.61	C27 CHCs	16.988; df = 2		2.0e-4
				pairwise: females M - females H		0	1.2e-4 [a]
				pairwise: females M - females L		37	0.80 [a]
				pairwise: females H - females L		80	1.6e-4 [a]

[a] Bonferroni-Holm corrected P-values

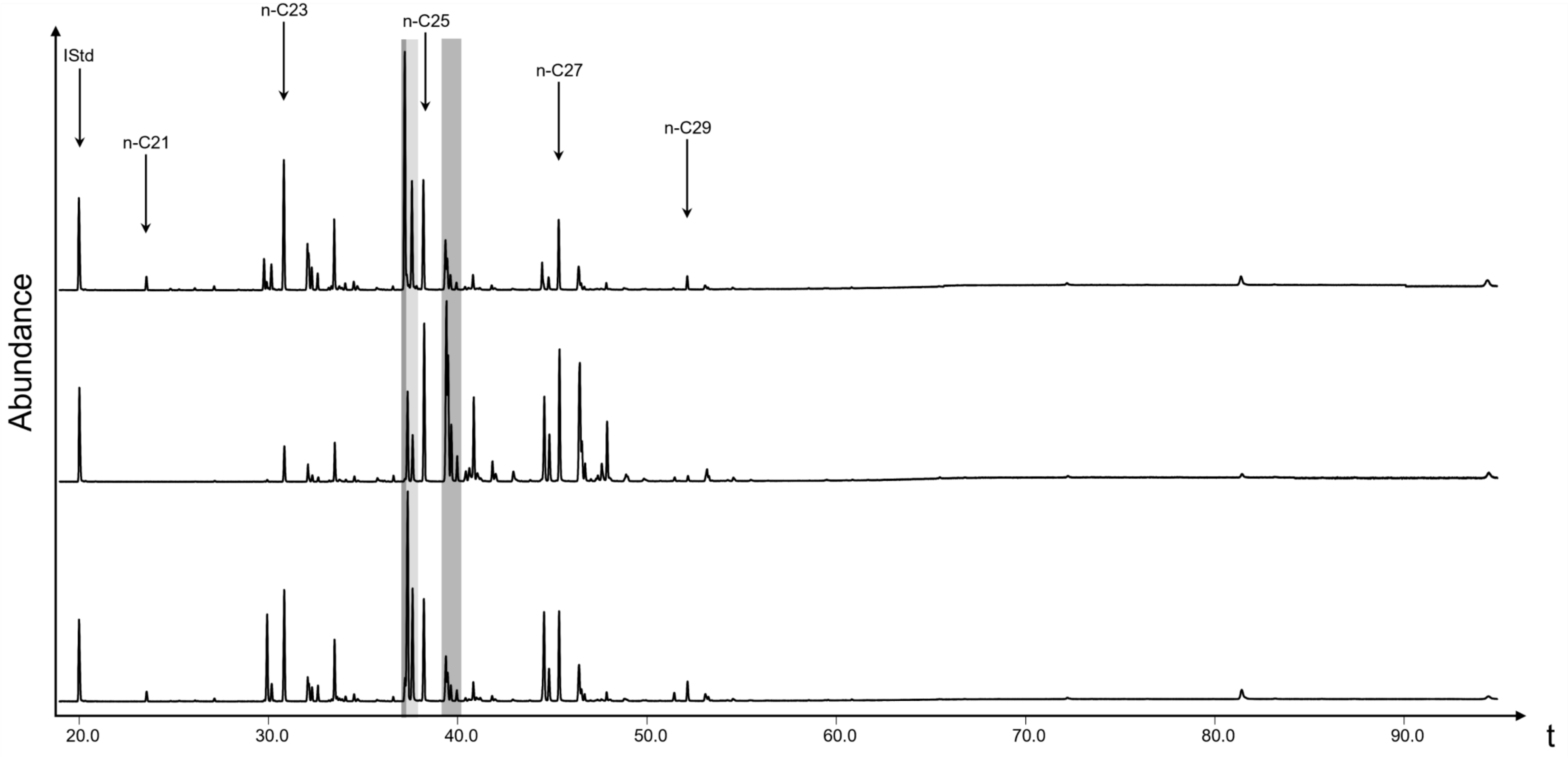

Figure S2: Chromatograms of female *T. zealandicus* showing the three chemotypes M (top), H (middle), and L (bottom) from the detailed analyses of wasps from chemotype pure matrilines. Alkanes are marked with arrows and named (Cx = chainlength of x carbon atoms). Patterns of hydrocarbons repeat for chain lengths of C23 to C29 (see Table S5). The most distinctive chromatogram for each chemotype was chosen to better illustrate the differences in relative abundances of alkadienes (example for 6,9-pentacosadiene marked with the first, dark grey underlay before n-C25), alkenes (example for pentacosenes marked by the second, light grey underlay before n-C25), and methylbranched alkanes (example for monomethyl pentacosanes marked by the grey underlay after n-C25). IStd = internal standard, 1-eicosene, 10ng/µl.
